# Potential Value of Serum Lipid in the Identication of Postoperative Delirium Undergoing Knee/Hip Arthroplasty: The Perioperative Neurocognitive Disorder and Biomarker Lifestyle Study

**DOI:** 10.3389/fpsyt.2022.870317

**Published:** 2022-04-12

**Authors:** Yanan Lin, Xiaoyan Peng, Xu Lin, Xiyuan Deng, Fanghao Liu, He Tao, Rui Dong, Bin Wang, Yanlin Bi

**Affiliations:** ^1^Department of Anesthesiology, Qingdao Municipal Hospital Affiliated to Qingdao University, Qingdao, China; ^2^Department of Anesthesiology, Dalian Medical University, Dalian, China; ^3^Department of Anesthesiology, Drum Tower Hospital Affiliated to Nanjing University Medical School, Nanjing, China

**Keywords:** delirium, triglycerides (TG), cholesterol, low-density lipoprotein (LDL), high-density lipoprotein (HDL), mediation effect

## Abstract

**Objective:**

We aimed to investigate the relationship between preoperative lipid level and postoperative delirium (POD) and explore whether lipid’s effect on POD is mediated by POD core protein.

**Methods:**

A total of 635 patients who were planned to undergo knee/hip arthroplasty under combined spinal-epidural anesthesia, regardless of gender, were selected. The patients were aged 40–90 years with American Society of Anesthesiologists physical status I II. The Mini-Mental State Examination (MMSE) was completed 1 day before the operation. Five milliliter elbow venous blood was taken from the patients before anesthesia, and serum levels of total cholesterol (TG), triglyceride (TC), low-density lipoprotein (LDL-C), and high-density lipoprotein (HDL-C) were detected. Cerebrospinal fluid (CSF) was extracted after successful spinal-epidural combined puncture, and amyloid beta_40_ (Aβ_40_), amyloid beta_42_ (Aβ_42_), total Tau (t-Tau), and phosphorylated Tau (p-Tau) in the CSF were measured by enzyme-linked immunosorbent assays (ELISA). After the operation, the occurrence and severity of POD were assessed using the Confusion Assessment Method and the Memorial Delirium Assessment Scale (MDAS), respectively. Patients were categorized into POD group and NPOD group. Logistic regression was used to analyze the relationship between POD and TC, TG, LDL-C, and HDL-C, and the mediating effect was used to analyze the role of POD core proteins in the relationship between lipid and MDAS. We used the receiver operating characteristic (ROC) and the precision-recall curve (PRC) analysis to assess the ability of TC, TG, LDL-C, and HDL-C ability to predict POD. Finally, we performed a sensitivity analysis to assess the stability of the results.

**Results:**

A total of 562 patients were finally enrolled in this study, and 66 patients developed POD, with an incidence of 11.7%. Logistic regression analysis showed that high concentration of TC (OR = 3.148, 95%CI 1.858∼5.333, *P* < 0.001), TG (OR = 2.483, 95%CI 1.573∼3.918, *P* < 0.001), and LDL-C (OR = 2.469, 95%CI 1.310∼4.656, *P* = 0.005) in serum were risk factors for POD. A high concentration of HDL-C (OR = 0.258, 95%CI 0.112∼0.594, *P* = 0.001) was a protective factor for POD after adjusted for age, sex, education, and MMSE score. ROC curves showed that HDL-C have the highest sensitivity and specificity in predicting POD. For these four lipid markers, the PRC range from 0.602 to 0.731, respectively. The mediating analysis showed that POD core proteins could partially mediate the relationship between lipid and POD (effect value: 16.19∼91.04%). The results were barely changed in the sensitivity analysis, and the sensitivity analysis has shown that the results were stable.

**Conclusion:**

The increase of serum TG, TC, and LDL-C concentration is a risk factor for POD development, while high HDL-C concentration is a protective factor for POD, and the occurrence of POD is caused by hyperlipidemia may be caused by POD core proteins.

**Clinical Trial Registration:**

[www.ClinicalTrials.gov], identifier [Chictr200033439].

## Introduction

Postoperative delirium (POD) is one of the most common complications in patients after surgery. POD may bring about a decline in cognitive ability, impair environmental interpretation, attention-deficit disorder, and dyssomnia (1). Moreover, it can ultimately lead to adverse outcomes such as an increased incidence of postoperative complications, more extended hospital stays, increased medical costs, and increased postoperative mortality (2). In consequence, it is of great importance to explore the effective forecasting methods for POD occurrence.

Metabolic disorder of blood lipids is also familiar in senior citizens. Some researchers found that hemorheology was associated with cognitive decline (3). Hyperlipidemia can lead to abnormal deposition of lipids in vascular endothelium and formation of atherosclerosis, damage the blood-brain barrier, resulting in abnormal accumulation of lipids in the brain, finally leading to the occurrence of neurodegenerative diseases. Changes in lipid-related metabolism and transport levels played a role in the prediction of Alzheimer’s disease (AD) (4), and plasma lipid metabolism levels in patients with cognitive impairment were also apparent differences from those in the normal subjects (5). Furthermore, elevated plasma triglyceride levels precede Amyloid-beta (Aβ) protein deposition (6), the value of triglyceride in predicting the occurrence of AD is not negligible. Hypercholesterolemia can exacerbate Aβ protein deposition in animal models (7), while in humans, lowering cholesterol levels can reduce the Aβ burden and reduce AD occurrence (8). A Retrospective cohort study shows that high cholesterol increases the risk of dementia (9). Aβ abnormal deposition is proportional to neurotoxicity (10, 11), abnormally phosphorylated tau protein deposited in cells to can form neurofibrillary tangles, which all can cause neurodegeneration finally (12). It is a neurodegenerative disease with AD, and delirium pathophysiology is similar to AD (13, 14). For the time being, however, there is still a lack of studies concerning whether Aβ and tau could modulate the relationships of hemorheology with POD.

Thus, we aimed to investigate the relevance between lipid levels and POD, test whether the influences of lipids on delirium were mediated by POD core pathology. All these analyses were conducted based on the Perioperative Neurocognitive Disorder and Biomarker Lifestyle (PNDABLE) study.

## Materials and Methods

### Participants

A total of 635 Han Chinese patients who were planned to undergo knee or hip arthroplasty under combined spinal-epidural anesthesia were selected from the PNDABLE study. The trial was carried out at Qingdao Municipal Hospital in Shandong Province, China. The PNDABLE study is an ongoing, large-sample cohort study that began in 2018 to explore the pathogenesis, risk factors, and biomarkers of perioperative neurocognitive dysfunction (PND) in the Han Chinese population in northern China for early detection, diagnosis, and intervention of PND. Cerebrospinal fluid (CSF) and blood samples were collected from all enrolled patients after they signed informed consent. The Ethics Committee (Ethical Committee N 2020 *PRO FORMA* Y number 005) approved this study of Qingdao Municipal Hospital.

We included the following patients: (1) The patients were aged 40 90 years old; (2) American Society of Anesthesiologists physical status(ASA)I∼II; (3) The patients had intact preoperative cognitive function without communication disorders; (4) The patients had sufficient education to complete the preoperative neuropsychological tests. Exclusion criteria included: (1) Mini-Mental State Examination (MMSE) scores of 23 or less; (2) ASA III or higher level; (3) Serious psychological disorders; (4) Severe systemic diseases that may affect related biomarkers in CSF or blood, including but not limited to malignant tumors; (5) Familial genetic diseases; (6) Coagulation dysfunction (possibly due to the long-term use of anticoagulants);

### Cognitive Measurements

The MMSE was used to evaluate the basic cognitive level of the patients the day before surgery. The Confusion Assessment Method (CAM) (15) was used to evaluate the postoperative cognitive level at 9:00–10:00 a.m. and at 2:00–3:00 p.m. twice a day on 1–7 days (or before discharge) by an anesthesiologist post-operatively. The diagnostic criteria for POD were as follows: ① acute changes and repeated fluctuations in the state of consciousness; ② lack of attention; ③ disorganized thinking; ④ alterations in the level of consciousness. CAM was determined to be positive if both ① and ② were present on any day, and at the same time either ③ or ④ was met. According to the assessment results, they were divided into the POD group and the NPOD group. Moreover, the POD severity was assessed using the MDAS (16).

### Anesthesia and Surgery

All the patients did not need any medication preoperatively. After the patients entered the operating room, peripheral veins were opened, and the same team of surgeons performed knee or hip arthroplasty. ECG, pulse blood oxygen saturation monitoring, and non-invasive arterial pressure measurement were routinely conducted. After the preparation was completed, the spinal and epidural anesthesia was performed in the lateral decubitus under L_3∼4_ space. After a successful puncture, 0.67% ropivacaine 2.0 ∼ 2.5 ml was injected into the subarachnoid space, and then 3–5 ml 2% lidocaine was added into the epidural catheter according to actual needs to maintain the level of anesthesia at T_8_ ∼ S_5_. If the intraoperative systolic blood pressure of the patient was < 90 mmHg, intravenous ephedrine 6 mg was given; If the patient’s heart rate was < 50 bpm, an intravenous injection of atropine 0.5 mg was given. Every patient was treated with a patient-controlled intravenous analgesia pump (Tropisetron 5 mg + Butorphanol Tartrate Injection 10 mg, diluted to 100 ml with normal saline at a rate of 2 ml/h) for 48 h postoperatively. After the operation, the patient was sent to the recovery room, observed for 30 min, and sent back to the ward if there was no abnormality. The duration of surgery, duration of anesthesia, intraoperative blood loss, and fluid input were recorded.

### Measurements of Cerebrospinal Fluid Sampling and Blood Sampling

After successful spinal-epidural anesthesia puncture, 2 ml of CSF was taken in 10 mL polypropylene tubes and sent to the laboratory within 2 h. The CSF samples were immediately centrifuged at 2,000 g at room temperature for 10 min and then stored at -80°C for further analysis. The levels of Aβ_40_, Aβ_42_, t-Tau and p-Tau in CSF were determined by enzyme-linked immunosorbent assays (ELISAs) on the microplate reader. CSF biomarkers of POD measurements were done with ELISA kits [Aβ_42_ (BioVendor, Ghent, Belgium Lot: No. 296-64401), P-tau (BioVendor, Ghent, Belgium Lot: QY-PF9092) and T-tau (BioVendor, Ghent, Belgium Lot: No. EK-H12242)]. All CSF samples were randomly distributed on the same batch of plates. All experimental procedures were performed by researchers who were blinded to patient information. All the antibodies and plates were from a single lot to exclude variability between batches. Moreover, the within-batch CV was < 5% and the inter-batch CV was < 15%.

After fasting for at least 8 h, the patient entered the operating room, and 5 ml of medial cubital vein blood was drawn. Venous blood was collected into vacuum tube, which was then measured by the hospital’s laboratory staff. Serum concentrations of total cholesterol (TC), triglycerides (TG), low-density lipoprotein (LDL-C), and high-density lipoprotein (HDL-C) were measured under standardized research protocols using an automatic biochemical analyzer (DURUI CS-600B, China).

### Statistical Analysis

SPSS statistical software, version 25.0 (SPSS, Inc., Chicago, IL, United States), and Medcalc software (version 20.0.1, Ostend, Belgium) were used for data analysis. Continuous variables were expressed as median and interquartile range (M, IQR), and compared using Mann-Whitney *U-*test. Categorical variables will be tested for baseline comparability with the chi-square test or Fisher’s exact test, expressed in frequency and percentage. To evaluate potential risk factors for POD, we used logistic regression analysis without and with adjustment for age, sex, education, and MMSE score. We also used the receiver operating characteristic (ROC) and the precision-recall curve (PRC) analysis to assess the ability of TG, TC, HDL-C, and LDL-C for predicting POD.

The mediation effect was also evaluated by PROCESS macro Version2.16.3. Statistical significance of the mediating effect was set at zero, which was not encompassed in the 95% CI. where each path of the model was controlled for age, sex, education, and MMSE score.

In addition, a sensitivity analysis was performed to assess the results stability. Sensitivity analysis was carried out as follows: First, we analyzed whether the association would change if only individuals who were aged over 65 at the baseline were selected; Secondly, we added more covariates, including self-reported history of type 2 diabetes (yes or no) and hypertension (yes or no).

The expected sensitivity was 80%, the expected specificity was 50%, and the allowable errors were all 0.05. Bilateral test was required, α was 0.05, and the missed visit ratio was calculated as 20%. The minimum sample size calculated by PASS software was 503.

## Results

### Participant Characteristics

**FIGURE 1:**
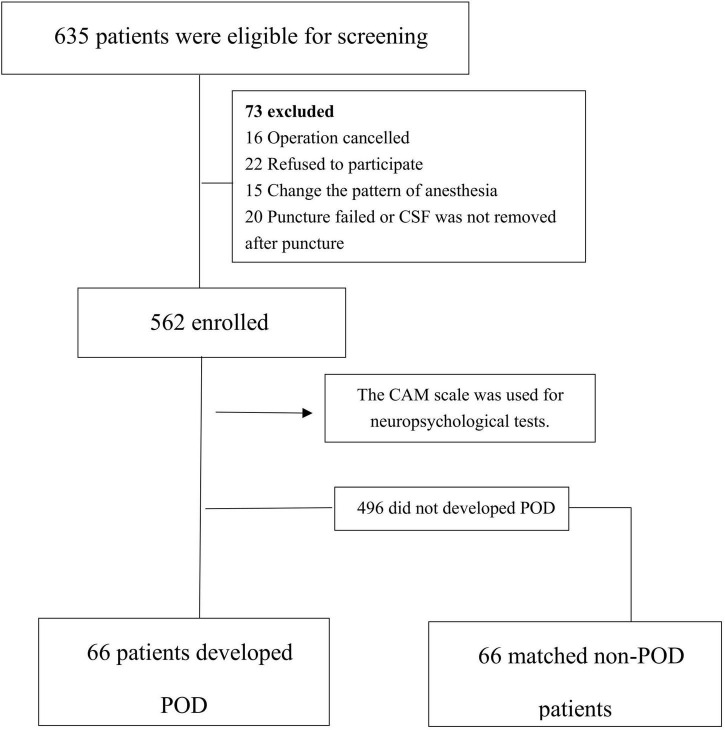
Flow diagram.

Among the 635 eligible patients, a total of 562 patients were finally included in this study. In the 562 patients, there were 66 POD cases, with an incidence of 11.7%, as shown in Figure 1. The incidence density sampling was used for the comparison between the POD group and the non-POD group, and 1:1 matching was performed on 5 variables, including ASA physical status, duration of surgery, duration of anesthesia, intraoperative blood loss, and fluid input.

The general conditions of the POD group and the NPOD group were compared (Table 1). There was no statistical significance in years of education, preoperative MMSE score, history of diabetes, or history of hypertension (*P* > 0.05), while the differences in sex, age, Serum TC, TG, LDL-C, HDL-C, CSF Aβ_40_, Aβ_42_, t-Tau, p-Tau, Aβ_42_/Aβ_40_, Aβ_42_/t-Tau, Aβ_42_/p-Tau, Aβ_40_/t-Tau, Aβ_40_/p-Tau, t-Tau/p-Tau, and Postoperative MDAS score were statistically significant (*P* < 0.05).

**TABLE 1:** Demographic and clinical characteristics.

Participant features	NPOD (*n* = 66)	POD (*n* = 66)	*P*
Male, *n* (%)	46(69.7%)	33(50%)	0.021
Age (year)	61(53.75−69.25)	68(57.00−71.00)	0.043
ASA physical status I, *n* (%)	58(87.9%)	51(77.3%)	0.108
History of hypertension, *n* (%)	21(31.8%)	30(45.5%)	0.108
History of diabetes, *n* (%)	11(16.7%)	16(24.2%)	0.281
Years of education (year)	12(9−14)	9(6−15)	0.392
Preoperative MMSE score	28(26.75−30)	28(25.75−29)	0.129
Serum TC (mmol/L)	4.59(3.89−5.00)	5.28(4.46−5.69)	<0.001
Serum TG (mmol/L)	1.26(0.97−1.77)	2.09(1.39−2.92)	<0.001
Serum HDL-C (mmol/L)	1.22(1.18−2.29)	1.18(1.01−1.39)	0.018
Serum LDL-C (mmol/L)	2.71(2.34−3.04)	2.76(2.51−3.34)	0.033
CSF Aβ_40_ (100 pg/mL)	37.35(26.86−49.74)	48.58(29.32−61.12)	0.032
CSF Aβ_42_ (pg/mL)	277.54(215.75−308.03)	142.57(114.29−184.02)	<0.001
CSF t-Tau (pg/mL)	167.02(141.30−252.79)	601.64(512.25−671.19)	<0.001
CSF p-Tau (pg/mL)	31.54(29.12−41.99)	82.03(75.84−90.05)	<0.001
CSF Aβ_42_/Aβ_40_	0.07(0.05−0.13)	0.03(0.02−0.05)	<0.001
CSF Aβ_42_/t-Tau	1.52(1.01−2.09)	0.24(0.19−0.34)	<0.001
CSF Aβ_42_/p-Tau	8.56(6.01−10.58)	1.81(1.42−2.26)	<0.001
CSF Aβ_40_/t-Tau	20.91(12.39−31.79)	7.94(4.73−11.79)	<0.001
CSF Aβ_40_/p-Tau	109.24(75.10−146.84)	58.51(35.32−82.11)	<0.001
CSF t-Tau/p-Tau	4.93(4.38−5.75)	7.22(6.29−8.26)	<0.001
Postoperative MDAS score	4.50(3.00−7.00)	18.00(17.00−20.00)	<0.001
Duration of surgery (min)	110(90−151.25)	110(90−138.75)	0.507
Duration of anesthesia (min)	170(135−200)	172.5(145−200)	0.417
Intraoperative blood loss (ml)	200(100−200)	200(130−200)	0.241
Intraoperative fluid input (ml)	1,100(712.5−1,100)	1,100(1,075−1,200)	0.290

*ASA, American Society of Anesthesiologists; MMSE, Mini-Mental State Examination; TC, triglyceride; TG, total cholesterol; LDL-C, low-density lipoprotein; HDL-C, high-density lipoprotein; MDAS, Memorial Delirium Assessment Scale.*

### Logistic Regression Analysis

Logistic regression analysis showed that high concentration of TC (OR = 3.148, 95%CI 1.858∼5.333, *P* < 0.001), TG (OR = 2.483, 95%CI 1.573∼3.918, *P* < 0.001), and LDL-C (OR = 2.469, 95%CI 1.310∼4.656, *P* = 0.005) in serum were risk factors for POD. A high concentration of HDL-C (OR = 0.258, 95%CI 0.112∼0.594, *P* = 0.001) was a protective factor for POD after adjusted for age, sex, education, and MMSE score (Table 2).

**TABLE 2:** Logistic regression analysis.

	Unadjusted			Adjusted*		
	*P*	OR	95% CI	*P*	OR	95% CI
Serum TC (mmol/L)	<0.001	2.584	1.633–4.089	<0.001	3.148	1.858–5.333
Serum TG (mmol/L)	<0.001	2.433	1.554–3.809	<0.001	2.483	1.573–3.918
Serum HDL-C (mmol/L)	0.001	0.271	0.124–0.590	0.001	0.258	0.112–0.594
Serum LDL-C (mmol/L)	0.012	2.111	1.177–3.787	0.005	2.469	1.310–4.656
CSF Aβ_40_ (100 pg/mL)	0.022	1.000	1.000–1.000	–	–	–
CSF Aβ_42_ (pg/mL)	<0.001	0.979	0.972–0.986	<0.001	0.979	0.971–0.986
CSF t-Tau (pg/mL)	<0.001	1.016	1.011–1.022	<0.001	1.021	1.012–1.031
CSF p-Tau (pg/mL)	<0.001	1.281	1.137–1.443	0.070	1.759	0.954–3.244
CSF Aβ_42_/Aβ_40_	<0.001	0.000	0.000–0.000	–	–	–
CSF Aβ_42_/t-Tau	<0.001	0.000	0.000–0.001	–	–	–
CSF Aβ_42_/p-Tau	<0.001	0.157	0.074–0.333	<0.001	0.142	0.061–0.332
CSF Aβ_40_/t-Tau	<0.001	0.825	0.769–0.884	<0.001	0.795	0.729–0.866
CSF Aβ_40_/p-Tau	<0.001	0.969	0.957–0.980	<0.001	0.966	0.953–0.978
CSF t-Tau/p-Tau	0.019	1.208	1.031–1.414	0.012	1.224	1.046–1.434

*The adjustment factors include age, sex, education, and MMSE score.

We performed two sensitivity analyses. In our first sensitivity analysis, we added more covariates, including self-reported history of type 2 diabetes and hypertension, and the results showed that high concentration of TC (OR = 3.394, 95%CI 1.953∼5.898, *P* < 0.001), TG (OR = 2.456, 95%CI 1.557∼3.872, *P* < 0.001) and LDL-C (OR = 2.650, 95%CI 1.376∼5.101, *P* = 0.004) in serum were remain risk factors for POD. After adjusted for age, sex, education, MMSE score, self-reported history of type 2 diabetes, and hypertension, high concentration of HDL-C (OR = 0.263, 95%CI 0.115∼0.601, *P* = 0.002) was a protective factor for POD (Supplementary Table 1). In the second sensitivity analysis, we selected patients older than 65 years old. The implication of these results is that high concentration of TC (OR = 3.880, 95%CI 1.653∼9.108, *P* = 0.002), TG (OR = 2.421, 95%CI 1.218∼4.809, *P* = 0.012) and LDL-C (OR = 2.639, 95%CI 1.032∼6.743, *P* = 0.043) in serum were remain risk factors for POD. After adjusted for age, sex, education and MMSE score, high concentration of HDL-C (OR = 0.163, 95%CI 0.040∼0.659, *P* = 0.011) was a protective factor for POD (Supplementary Table 2). The results were barely changed in the sensitivity analysis, and the sensitivity analysis have showed that the results were stable.

### Receiver Operating Characteristic Analysis and Precision-Recall Curve Analysis

**FIGURE 2:**
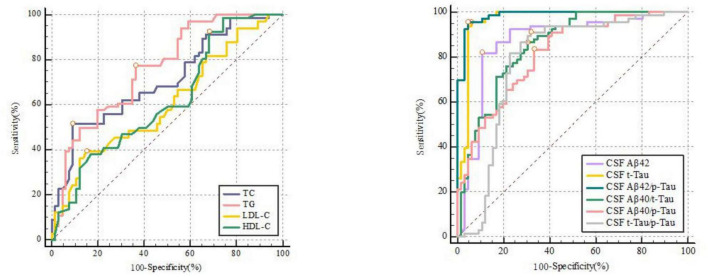
The receiver-operator characteristic analyses for TC [0.708 (0.623–0.784)], TG [0.761 (0.679–0.831)], LDL-C [0.607 (0.519–0.691)], HDL-C [0.620 (0.531–0.703)] and CSF biomarkers in predicting delirium.

**FIGURE 3:**
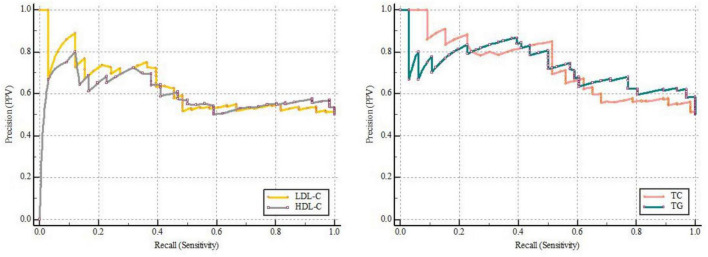
The precision-recall curve of predicting postoperative delirium.

ROC curves showed that LDL-C [0.607 (0.519–0.691)], HDL-C [0.620 (0.531–0.703)], TG [0.761 (0.679–0.831)], and TC [0.708 (0.623–0.784)] can all predict POD (Figure 2 and Supplementary Table 3). Among which, HDL-C had the highest sensitivity and specificity in predicting POD, although AUC was not the largest. We calculated the area under curve and F1 score of TG, TC, LDL-C, and HDL-C in PRC analysis. The results showed that these four lipid markers, the PRC range from 0.602 to 0.731, respectively. The F1 score of TG, TC, LDL-C, and HDL-C were 0.757, 0.714, 0.685, and 0.722, respectively (Figure 3 and Supplementary Table 4).

### Mediation Analyses

**FIGURE 4:**
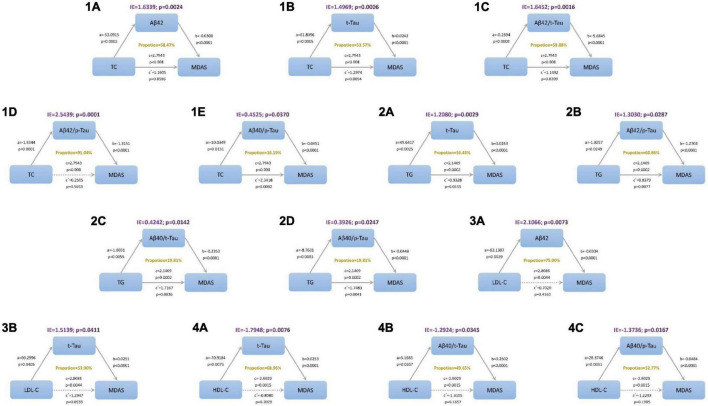
Mediation analyses with Memorial Delirium Assessment Scale (MDAS) as outcome. The relationship between triglyceride (TC) and postoperative delirium severity was mediated by amyloid and tau pathology indicated by (1A) amyloid beta_42_ (Aβ_42_), (1B) total Tau (t-Tau), (1C) amyloid beta_42_/t-Tau (Aβ_42_/t-Tau) ratios, (1D) Aβ_42_/p-Tau ratios, (1E) Aβ_40_/p-Tau ratios. The relationship between total cholesterol (TG) and postoperative delirium severity was mediated by amyloid and tau pathology indicated by (2A) t-Tau, (2B) Aβ_42_/t-Tau ratios, (2C) Aβ_40_/t-Tau ratios, (2D) Aβ_40_/p-Tau ratios. The relationship between low-density lipoprotein (LDL-C) and postoperative delirium severity was mediated by amyloid and tau pathology indicated by (3A) Aβ_42_ and (3B) t-Tau. The relationship between high-density lipoprotein (HDL-C) and postoperative delirium severity was mediated by amyloid and tau pathology indicated by (4A) t-Tau, (4B) Aβ_40_/t-Tau ratios and (4C) Aβ_40_/p-Tau ratios. IE, indirect effect.

In the mediation modeling analysis, we assessed the mediation effects of CSF proteins on the associations of lipid levels with MDAS, after controlling for age, sex, education, and MMSE score. The relationship between TC and POD severity was mediated by amyloid and tau pathology indicated by Aβ_42_, t-Tau, Aβ_42_/t-Tau ratios, Aβ_42_/p-Tau ratios, and Aβ_40_/p-Tau ratios. While, the relationship between TG and MDAS was mediated by t-Tau, Aβ_42_/t-Tau ratios, Aβ_40_/t-Tau ratios, and Aβ_40_/p-Tau ratios. Aβ_42_ and t-Tau act as full mediators between LDL-C and MDAS. The result of this study shows that t-Tau, Aβ_40_/t-Tau ratios, and Aβ_40_/p-Tau have different performance on HDL-C and MDAS (Figure 4).

## Discussion

As far as we are aware, the study is the first that reported the relationship including mediator effects between serum lipid and POD. We mainly screened out several POD core proteins as mediators. Of course, they played different mediating effects in the relationship between different serum lipoprotein and POD.

Cholesterol is an essential component of membranes and plasma lipoprotein, and it also plays an essential part in the accommodation of synaptic function and cell plasticity (17). An independent study (18) found that hypercholesterolemia caused memory impairment, inflammation response, and cholinergic dysfunction. Conversely, taking cholesterol-reducing medications can bring down the risk of neurocognitive-related diseases (19). Our findings indicate that cholesterol amounts altered was concerned with POD, and serum cholesterol was proportional to the severity of POD; that is, it is positively correlated with MDAS scores. Hypercholesterolemia leading to POD partly by Aβ_42_, t-Tau, Aβ_42_/t-Tau ratios, Aβ_42_/p-Tau ratios, and Aβ_40_/p-Tau ratios, explanation by mediation effects. Likewise, Umeda et al. found that hypercholesterolemia accelerates the accumulation of Aβ oligomers and resulting in memory impairment (20). It is universally recognized that reduced CSF Aβ_42_ concentration reflects the accumulation of aggregated Aβ_42_ in amyloid plaques in the brain (21). In patients with hip fracture, this group found lower CSF Aβ_42_ levels and increased CSF t-Tau levels who developed delirium compared to the control group, the biomarkers remained significant after adjusting for age, gender, and Informant Questionnaire on Cognitive Decline in the Elderly score. This result is consistent with our findings. Some research has found that cholesterol amounts modification altered amyloid precursor protein (APP) and Aβ expression (22, 23). Cholesterol transcellular transportation was altered by Aβ, while inhibition of intracellular transport of cholesterol reduced cleavage of Aβ from APP in neurons (24, 25). Intracellular cholesterol plays a significant role in modulating tau phosphorylation and maintaining microtubule stability, the researchers found (26). Van der et al. (27) found that the effects of cholesterol on tau proteostasis are correlated with APP and Aβ. We also find this relationship by calculating the mediating effect. The interesting thing is that exercise can lower the tau pathology and its pathophysiological consequences (28). Exercise decreased the levels of soluble Aβ_40_ and Aβ_42_ (29), also reducing the lipid level in serum. It is tempting to think there is at least a case to be made for exercise to lower cholesterol levels and thus reduce the risk of POD.

Moreover, our results showed that triglyceride levels were higher than the NPOD group in POD patients, and the difference between the two groups has statistical significance. t-Tau, Aβ_42_/t-Tau ratios, Aβ_40_/t-Tau ratios, and Aβ_40_/p-Tau ratios may mediate the effect of triglyceride on POD. Triglyceride components were found to be significantly associated with CSF Aβ_42_ values (30). A longitudinal cohort study in cognitively healthy individuals concluded that increased levels of triglycerides could even predict CSF Aβ and tau pathology 20 years later (31). Higher serum triglyceride levels are associated with Parkinson’s disease mild cognitive impairment (32) and are one of the risk factors for AD (33). It was proved that triglycerides could cross the blood-brain barrier (BBB), consisting of human CSF, resulting in cognitive impairment (34). Some scholars have argued that the relation between triglycerides and cognition may be mediated by triglyceride regulation of the BBB transport of cognitively active gastrointestinal hormones (35). In animal models, an influential study showed that plasma triglyceride levels increased precede Aβ deposition (6), but total cholesterol levels were not significantly different in this research. In another model of hyperlipidemia-induced age-related neurodegeneration (36), chronic hypertriglyceridemia may lead to impaired neuronal function and neurodegeneration, possibly via hyperphosphorylation of tau protein, and this is similar to our findings.

More importantly, our analysis found that serum HDL level is associated with POD development, and high serum HDL level before surgery is one of the protective factors of POD. HDL-C is known as the “good cholesterol” because of its ability to reverse cholesterol transport. It protects against elevated lipid levels and protects against endothelial dysfunction, oxidative stress, inflammation, thrombosis, and more. Therefore, it is well known that serum HDL-C level is associated with a lower risk of cardiovascular disease. In addition, several studies have shown that individuals with higher levels of serum HDL-C is related to better cognitive function status (37–39), One possible reason is that HDL-C is capable of binding Aβ (40) and prevent Aβ aggregation into amyloid (41), and then improve clearance of Aβ from the brain, which in turn decreases the neurotoxicity of Aβ peptides (42). Another factor may be that serum HDL-C levels are inversely correlated with brain Aβ deposits (43). Our study did not support a significant mediation effect of Aβ deposits in the associations between serum HDL-C and MDAS, while the t-Tau, Aβ_40_/t-Tau ratios, and Aβ_40_/p-Tau ratios play full mediators on the relationship between HDL-C and MDAS. A study of older adults in China’s rural area showed that low HDL-C is associated with structural brain aging and cognitive dysfunction, but the association of low HDL-C with cognitive aging is not mediated by brain structure (44). Our data agree with previous research that low HDL-C is associated with cognitive impairment and dementia and is a risk factor for memory deficit and decline (45).

Our data insinuate that preoperative LDL-C levels were positively correlated with POD occurrence. Aβ_42_ and t-Tau may mediate the effect of LDL-C on POD. In addition, Aβ_42_ is a complete mediation. Our data support the view that a higher LDL-C level was associated with higher Aβ deposition and lower cognitive function (46, 47). In an Australian study, researchers discovered that higher levels of cholesterol and LDL-C were related to impaired processing speed, recognition memory, and working memory (48). However, in a prospective cohort study in Japan, higher LDL-C levels were associated with higher scores in memory performance after controlling for confounders (49), The Japanese study is broadly similar to the results of a cross-sectional study from China (50). According to the Chinese study, higher LDL-C was significantly negatively related to higher MMSE scores among the oldest old (aged 80 + years). Another Chinese study showed that a high level of LDL-C may be considered a potentially protective factor against cognition decline (51). Still, some research showed that LDL-C level did not influence the incidence of cognitive disorder or global cognitive performance (52, 53). All of the above studies come from different countries and regions, with different living standards and educational levels, so many factors influence the results. Therefore, future large-sample multicenter studies are needed to support the relationship between LDL-C and POD.

There are limitations to this study. As this is an observational cross-sectional design, we only tried to infer the causal relationship, but the specific relationship needs further study. In addition, our study only measured lipid levels at a one-time point before surgery, and more comprehensive monitoring of lipid levels is needed in the future. The research population we included come from the same hospital, which is also the deficiency of our experimental study. If possible, we hope to conduct verification of our experimental model in other independent and comparable hospitals in future studies.

To sum up, the present study indicated that the increase of serum TG, TC, and LDL-C concentration are risk factors for the development of POD, while high HDL-C concentration is a protective factor for POD, and the occurrence of POD caused by hyperlipidemia may be caused by POD core protein. Therefore, we advocate maintaining a healthy lifestyle to reduce lipid levels and thus reduce the incidence of POD.

## Data Availability Statement

The original contributions presented in the study are included in the article/Supplementary Material, further inquiries can be directed to the corresponding author/s.

## Ethics Statement

The studies involving human participants were reviewed and approved by the Clinical Trial Ethics Committee of Qingdao Municipal Hospital. The patients/participants provided their written informed consent to participate in this study.

## Author Contributions

YL contributed to the statistical analysis, and manuscript preparation. XD, FL, and HT involved in the data collection and ELISA performance. XP, XL, and RD revised the manuscript. YB and BW conceived the current study. All authors have contributed to the manuscript revising and editing critically for important intellectual content and given final approval of the version and agreed to be accountable for all aspects of the work presented here, reviewed, and approved the final manuscript.

## Conflict of Interest

The authors declare that the research was conducted in the absence of any commercial or financial relationships that could be construed as a potential conflict of interest.

## Publisher’s Note

All claims expressed in this article are solely those of the authors and do not necessarily represent those of their affiliated organizations, or those of the publisher, the editors and the reviewers. Any product that may be evaluated in this article, or claim that may be made by its manufacturer, is not guaranteed or endorsed by the publisher.
