# Supplementary figures and images for: Potential Value of Serum Lipid in the Identication of Postoperative Delirium Undergoing Knee/Hip Arthroplasty: The Perioperative Neurocognitive Disorder and Biomarker Lifestyle Study
Source: Front Psychiatry. 2022 Apr 12;13:870317. doi: 10.3389/fpsyt.2022.870317 (PMC9039337; doi:10.3389/fpsyt.2022.870317)

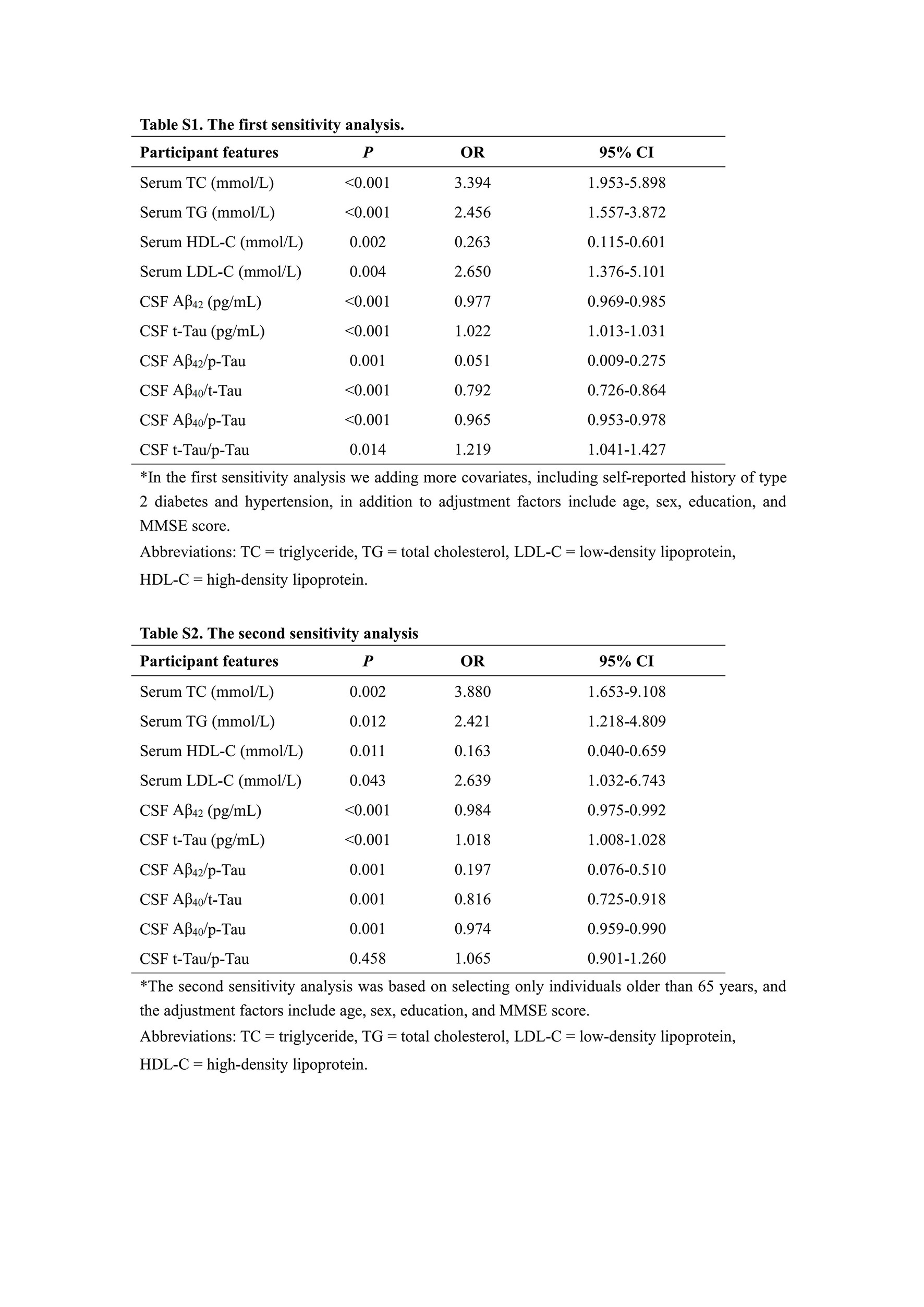

Supplement: Supplementary file 1 [file Image_1.JPEG]

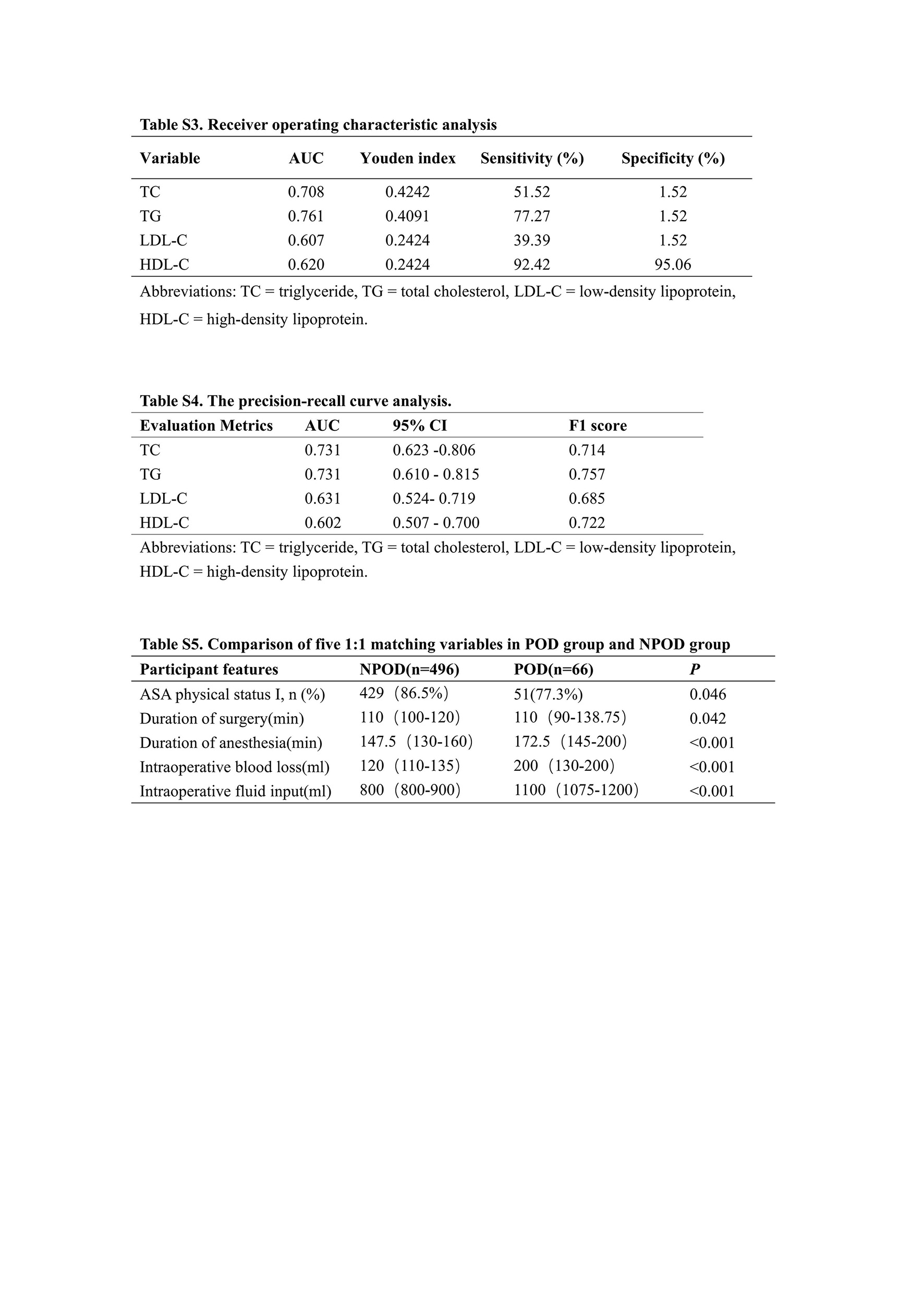

Supplement: Supplementary file 2 [file Image_2.JPEG]
